# The value of melatonin supplementation in postmenopausal women with *Helicobacter pylori*-associated dyspepsia

**DOI:** 10.1186/s12905-020-01117-z

**Published:** 2020-11-26

**Authors:** Cezary Chojnacki, Marta Mędrek-Socha, Paulina Konrad, Jan Chojnacki, Aleksandra Błońska

**Affiliations:** Department of Clinical Nutrition and Gastroenterological Diagnostics of Medical University, Lodz, Poland

**Keywords:** Menopause, Dyspepsia, *Helicobacter pylori*, Melatonin

## Abstract

**Background:**

Dyspeptic syndrome is particularly common in postmenopausal women in the form of epigastric pain. The aim of the study was to assess the role of melatonin in chronic dyspepsia in this group of women, and examine the role of Helicobacter infection.

**Methods:**

The study comprised 152 subjects including 30 healthy women (Group I), 60 women with asymptomatic *H.pylori* infection (Group II), and 64 women with *H. pylori* infection with chronic dyspepsia (Group III). Endoscopic examination was performed, as well as histological assessment of gastric end duodenal mucosa, urease breath test (UBT-13C), and immunoenzymatic assessment of serum 17-β-estradiol, follicle stimulating hormone and melatonin, and urinary 6-sulfatoxymelatonin. In Group III, 14-day antibacterial treatment was introduced with pantoprazole, amoxicillin and levofloxacin followed a six-month treatment with placebo in 32 women (Group IIIa), and melatonin 1 mg/morning and 3 mg/at bedtime in the other 32 women (Group IIIb).

**Results:**

No significant differences were found between serum level of female hormone. Serum melatonin levels were similar between Group I (12.5 ± 2.72 pg/ml) and Group II (10.5 ± 3.73 pg/ml; *p* > 0,05). The level was significantly lower in Group III (5.72 ± 1.42 pg/ml; *p* < 0.001). Eradication of *H.pylori* was obtained in 75.0% women in Group IIIa, and in 84.3% in Group IIIb (*p* > 0.05). After six months, dyspeptic symptoms resolved in 43.7% patients in Group IIIa and 84.3% in Group IIIb (*p* < 0.001).

**Conclusion:**

Melatonin supplementation is useful in treating *H. pylori*-associated dyspepsia, particularly in postmenopausal women with lower levels of this hormone.

**Trial registration:**

NCT04352062, date of registration: 15.04.2020.

## Background

Following the menopause, many women can demonstrate range of psychosomatic disorders. The severity of climacteric symptoms, typically hot flushes, excessive sweating, sleep disorders, irritability, depressive mood, fatigue, headache, vertigo, myalgia, palpitation and formication, can be assessed using the Menopause Rating Scale [1]. However, this index does not take into account the gastrointestinal disorders which frequently cause chronic dyspepsia. Dyspeptic syndrome, in the form of epigastric pain, hunger, nocturnal pain and appetite disorders is particularly frequent. These symptoms are caused by a fall in estrogen level, which exerts a protective effect by inhibiting the secretion of hydrochloric acid and pepsin [2] and motor activity [3], as well as stimulating the secretion of mucus and bicarbonates [4]. Furthermore, estradiol exerts antioxidant activity [5, 6] and modulates visceral sensation [7].

However, dyspeptic problems are not relieved by hormone replacement therapy [8]. This may be due to the fact that in addition to estrogens, the postmenopausal period is characterized by a deficiency of various hormones, including melatonin [9, 10]. Experimental studies have shown that like estrogens, melatonin has an inhibitory effect on the secretion of hydrochloric acid [11] and stimulates the secretion of bicarbonates in the upper gastrointestinal tract [12]. It also demonstrates antioxidant [13], cytoprotective [14], myorelaxant [15] and analgesic [16] properties. Simultaneous estrogen and melatonin deficiency may create adverse conditions in the stomach and trigger dyspeptic discomfort. This assumption is supported by the results of earlier studies, which found reduced melatonin secretion in patients with functional dyspepsia [17]. The expression of melatonin-synthesizing enzymes in the gastric mucosa [18, 19] and the concentration of melatonin in gastric juice [20] may also be reduced.

The aim of the present study was to assess the role of melatonin in the pathogenesis of chronic dyspepsia in postmenopausal women, and to examine the effect of *Helicobacter pylori* infection.

## Methods

The study comprised 152 women aged 49–64 years (mean age 56.3 ± 8.3 years). The group included 62 women who developed dyspeptic problems for the first time after menopause. The study took place in the years 2011–2018.

Three groups were distinguished: Group I—30 women without dyspeptic complaints and without *Helicobacter pylori* infection; Group II—60 women with asymptomatic *Helicobacter pylori* infection; Group III—62 women with chronic dyspepsia and *Helicobacter pylori* infection.

Diagnosis of *H. pylori*—associated dyspepsia was based on the Kyoto Global Consensus [21].

### Inclusion criteria

The main symptoms reported in Group III were epigastric pain of a hunger nature and pain at night, as well as increased appetite. The severity of dyspeptic symptoms was evaluated using the Visual Analogue Scale. All subjects underwent endoscopic examination of the upper gastrointestinal tract and histological assessment was performed using hematoxylin–eosin and Giemsa staining. To confirm *Helicobacter pylori* infection, the UBT-13C urea breath test was performed using FANci-2 System (Fisher Instrumente, GmbH, Hamburg, Germany).

### Exclusion criteria

The following women were excluded from the study: those with other functional or inflammatory diseases of the gastrointestinal tract, liver and pancreas; those with metabolic, allergic or mental illness; those who were receiving hormone replacement therapy.

### Laboratory tests

The following routine laboratory examinations were performed: blood cell count, C-reactive protein, glycosylated hemoglobin, bilirubin, alanine and aspartate aminotransferase, amylase, lipase, urea, creatinine, cholesterol HDL and LDL, triglyceride assay.

Immunoenzymatic assay of 17-β-estradiol (antibodies Ortho-Clinical Diagnostics, Inc., Raritan, NY, USA) and follicle-stimulating hormone (FSH—Vitros Product antibodies—Ortho-Clinical Diagnostics, Inc.,Rochester, NY, USA) was also performed. Serum melatonin level and urinary 6-sulfatoxymelatonin level were measured by ELISA with IBL antibodies (RE-54021 and RE-54031, IBL International GmbH, Hamburg, Germany) and Expert 99 MicroWin 2000 Reader (GmbH, Labtech, Offenburg, Germany).

Blood samples were drawn from the antecubital vein at 9:00 a.m. and were frozen at minus 70°C. On the same day, samples of urine were taken over 24 h. Next morning 20 ml volume of urine samples were frozen at minus 70°C.

The subjects followed the same diet for seven days prior to the evaluations. On the day of the study, all patients consumed the same liquid diet (Nutridrink—Nutricia), containing 18.9 g carbohydrate, 6.0 g protein and 5.8 g lipid per ml. Three 400 ml meals were taken, with a total caloric value of 1800 kcal, together with 1500 ml of isotonic water.

### Therapeutic procedure

In group III, the following 14-day antibacterial treatment was introduced: pantoprazole (2 × 40 mg), amoxicillin (2 × 1000 mg) and levofloxacin (2 × 500 mg).

Afterward, the patients were randomly divided into two equally-sized groups. Group IIIa (n = 32) was administered placebo (LEK–KAM, Poland) as two tablets a day, and Group IIIb (n = 32) received melatonin at a dose of 1 mg/morning and 3 mg/at bedtime, for six months. In this period, the patients applied the same balanced diet with a total caloric value of 1600 kcal.

Follow-up clinical examinations were performed after one, three and six months, and the UBT-13C test was performed after three and six months.

### Statistical analysis

Normally distributed data was compared using Student’s t-test, and non-normal data by the Kruskal–Wallis and post hoc tests. Data were expressed as mean and standard deviation. Therapeutic effects after melatonin supplementation were evaluated using the chi-square test. A *p* value of < 0.05 was considered statistically significant. Statistica 13.3 (StatSoft, INC, USA) and MS Excel (Microsoft Co., USA) were used for statistical calculations.

## Results

The general characteristics of the investigated women are shown in Table 1. The groups did not differ in terms of age, body mass index, aminotransferase levels or renal filtration index. However, the result of the UBT-13C test excluded *H. pylori* infection in the control group.

**Table 1:** The general characteristics women included in the study: Group I—healthy women; Group II—postmenopausal women with asymptomatic Helicobacter pylori infection; Group III—postmenopausal patients with symptomatic *H. pylori* infection

Feature	Group I (n = 30)	Group II (n = 40)	Group III (n = 64)
Age (years)	54.6 ± 7.2	57.4 ± 8.2	56.8 ± 7.9
BMI (kg/m^2^)	23.8 ± 1.6	24.1 ± 2.3	25.6 ± 6.1
UBT—13 C (ppm)	–	18.4 ± 4.6	21.0 ± 6.1
ALT (IU/L)	21.6 ± 6.2	26.3 ± 4.4	24.2 ± 6.1
AST (IU/L)	20.6 ± 4.0	25.1 ± 3.8	22.8 ± 6.9
GFR (ml/min)	98.5 ± 11.8	97.6 ± 12.1	102.4 ± 11.3

*BMI* body mass index, *UBT-13C* urease breath test, *ALT* alanine aminotransferase, *AST* aspartate aminotransferase, *GFR* glomerular filtration rate; differences between groups no statistically significant, *p* > 0.05

**Fig. 1:**
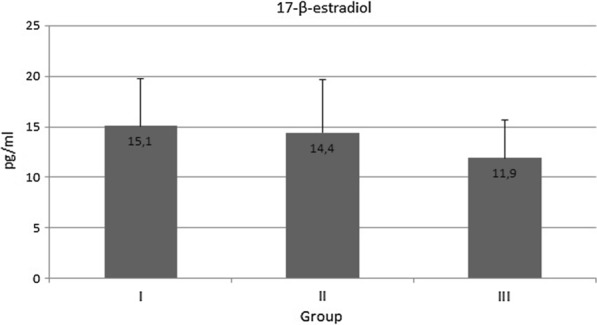
The serum level of 17-β-estradiol in healthy women (Group I), in women with asymptomatic *H. pylori* infection (Group II) and in women with both *H. pylori* infection and chronic dyspepsia (Group III); no significant differences were observed between groups (*p* > 0.05)

The serum levels of 17-β-estradiol were 15.1 ± 4.64 pg/ml in Group I, 14.4 ± 5.27 pg/ml in Group II and 11.9 ± 3.72 pg/ml Group III. These differences were not statistically significant (Fig. 1).

**Fig. 2:**
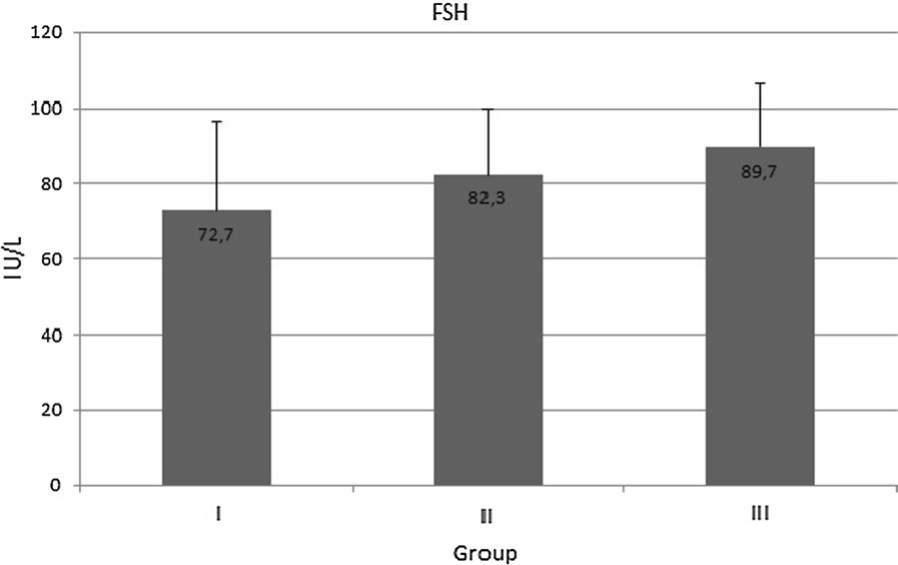
The serum level of follicle-stimulating hormone FSH) in healthy women (Group I), in women with asymptomatic *H. pylori* infection (Group II), and in women with both *H.pylori* infection and chronic dyspepsia (Group III); no significant differences were found between groups (*p* > 0.05)

Similarly, no significant differences in serum follicle-stimulating hormone levels were found between the groups: Group I—72.7 ± 23.6 IU/ml; Group II—82.3 ± 17.5 IU/ml; Group III—89.7 ± 16.9 IU/ml (Fig. 2).

**Fig. 3:**
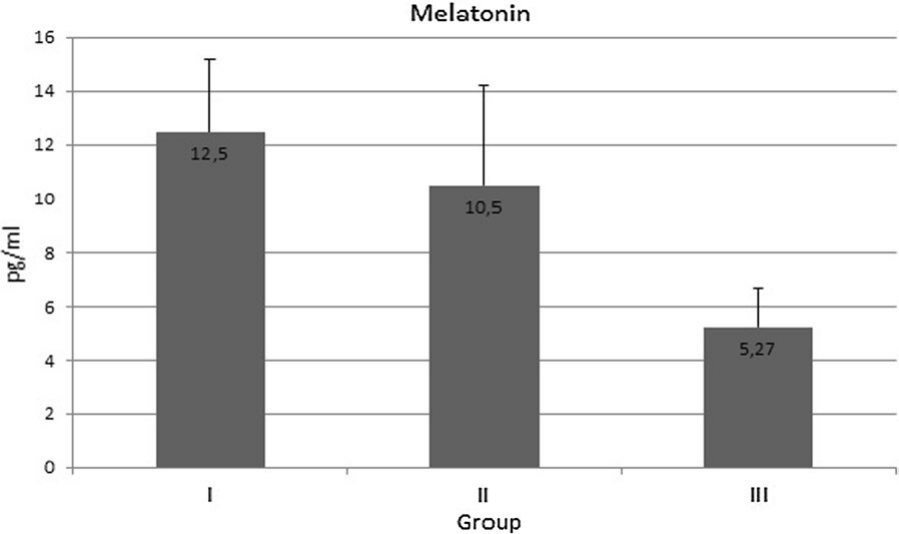
The serum level of melatonin in healthy women (Group I), in women with asymptomatic *H. pylori* infection (Group II), and in women with both *H. pylori* infection and chronic dyspepsia (Group III); differences between groups I and II (*p* > 0.05), I and III (*p* < 0.001), II and III (*p* < 0.001)

Similar serum melatonin levels were observed in Group I (12.5 ± 2.72 pg/ml) and Group II (10.5 ± 3.73 pg/ml; *p* > 0.05). However lower levels were found in women with symptomatic *H. pylori* infection: 5.27 ± 1.42 pg/ml (*p* < 0.001, Fig. 3).

**Fig. 4:**
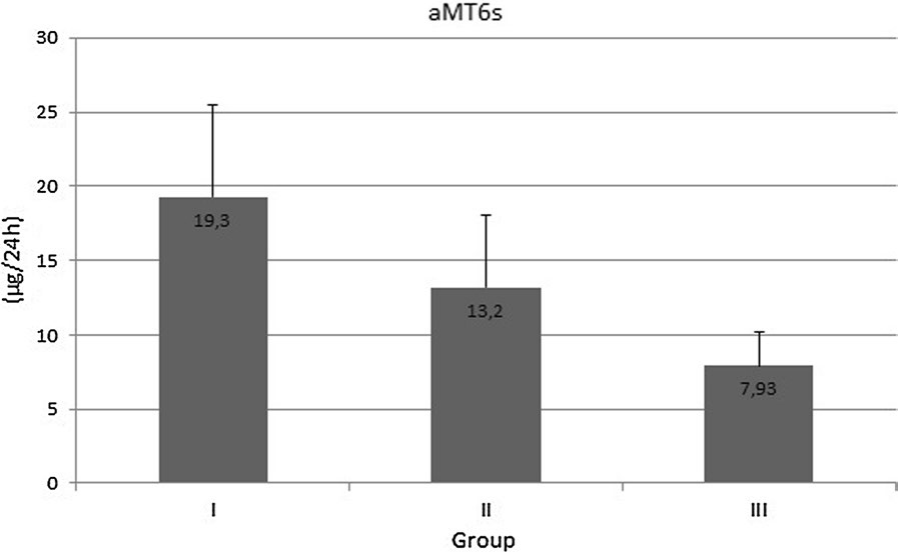
Urinary 6-sulfatoxymelatonin excretion in healthy women (Group I), in women with symptomatic *H. pylori* infection (Group II), and in women with both *H. pylori* infection and chronic dyspepsia (Group III); differences between all groups are statistically significant (*p* < 0.001)

Interestingly, significant differences were observed between the groups with regard to urinary 6-sulfatoxymelatonin excretion over 24 h: 19.3 ± 6.18 µg in Group I, 13.2 ± 4.80 µg in Group II—(*p* < 0.001), and 7.93 ± 2.27 µg/ml in Group III (*p* < 0.001, Fig. 4).

Eradication of *Helicobacter pylori* was obtained in 24 women (75.0%) in Group IIIa, and in 27 women (84.3%) in Group IIIb (*p* > 0.05).

After six months, dyspeptic symptoms resolved in 14 women (43.7%) in Group IIIa, and in 27 (84.3%) in Group IIIb (*p* < 0.001, Table 2).

**Table 2:** The results of *Helicobacter pylori* eradication and dyspeptic symptom improvement in patients receiving placebo (Group IIIa) or melatonin (Group IIIb)

Patients	Group IIIa N = 32		Group IIIb N = 32		χ^2^—value	*P* value
	n	%	N	%		
Without *H.pylori*						
3 months	24	75.0	26	81.2	0.366	0.545
6 months	23	71.8	27	84.3	1.459	0.227
Without symptoms						
3 months	12	37.5	16	50.0	1.014	0.314
6 months	14	43.7	27	84.3	11.489	0.0007

Melatonin was well tolerated: only four women (12.5%) reported increased fatigue in the morning, and two patients (6.2%) headache in the first week of the treatment. No cases required discontinuation of therapy or dose reduction.

## Discussion

In humans, melatonin secretion decreases with age [22]. These changes are particularly apparent in perimenopausal women [23, 24]. Some researchers believe that the reduction in melatonin secretion in women begins around the age of 40 years and may initiate menopause [25]. In this period of life, dyspeptic symptoms are frequently observed. The obtained results indicate that lowered melatonin levels may be one of reason of dyspeptic symptoms. Interestingly, while women with relatively normal melatonin levels tended to demonstrate asymptomatic *H. pylori* infection, those with both *H. pylori* infection and with low melatonin levels were more likely to suffer from dyspepsia. In the latter cases, there were indications for antibiotic therapy; however, eradication of *H. pylori* only eliminated complaints in some patients. Hence, it appears that dyspeptic symptoms may be associated with low secretion of melatonin in the gastric mucosa, and melatonin may have a protective effect in asymptomatic infections. Many studies have shown that even asymptomatic infection induces destructive changes in the gastric mucosa [26, 27] and the presence, or absence, of symptoms depends on many factors. The presence of gastrotoxic factors and absence of enteroprotective factors can trigger dyspeptic symptoms and can hasten the development of peptic ulcers and stomach cancer [28, 29].

Melatonin demonstrates many beneficial effects in the combined therapy of many gastrointestinal diseases, such as esophageal reflux disease [30], functional dyspepsia [31],ulcer disease [32], irritable bowel syndrome [33, 34] and ulcerative colitis [35, 36]. However, the optimal doses needed for therapeutic effectiveness and good tolerance remain unknown. A review of 392 previous studies found that the applied dose ranged from 0.3 mg to 1000 mg/daily [37], while another indicated from 0.1 to 50 mg/daily [38]. In order to control sleep, the most frequently recommended dose was 1 mg to 5 mg at night, while most treatments of alimentary tract disease [30–35] or climacteric disorders in women were based on doses of 3 mg or 5 mg per day [39, 40]. A dose of 8 mg per day was found to be an effective treatment of metabolic syndrome [41], and a dose of 50–100 mg daily has been proposed for the regulation of inflammatory and metabolic disorders [42].

Melatonin demonstrates good tolerability and safety, due to its pharmacokinetic properties. Oral administration of 10 mg melatonin resulted in a maximum serum concentration of 3550 pg/ml at T_1/2_ 53.7 min [43]. Similar results pharmacokinetics were obtained by other researchers following oral doses of 0.4 mg or 4 mg [44] and 80 mg [45]. Thus, the administration of a single dose of melatonin raises its serum level for a few hours. Therefore, to take the best advantage of its effect in postmenopausal disorders, melatonin should be administered in divided doses (1 mg/morning and 3 mg/at bedtime); nevertheless, its dose should be related to age, severity of symptoms and concomitant disease.

Our study has same limitations. The most significant one was the small size of the study group; even so, the population was relatively homogeneous and well characterized.

## Conclusion

Melatonin supplementation can play a significant role in complex therapy of *H. pylori*-associated dyspepsia, particularly in patients with reduced melatonin secretion, such as postmenopausal women.

## Data Availability

All data is available from the corresponding author on reasonable request.

## Abbreviations

aMT6s: 6- Sulfatoxymelatonin
BMI: Body Mass Index
ALT: Alanine aminotranseferase
AST: Aspartate aminotranxferase
FSH: Follicle-stimulating hormone
GFR: Glomerular filtration rate
UBT-13C: Urease breath test

## References

[1] Schneider HP, Heinemann LA, Rosemeier P, Potthoff P, Behre HM (2000). The Menopause Rating Scale (MRS): reliability of scores of menopausal complaints. Climacteric..

[2] Amure BO, Omole AA (1970). Sex hormones, and acid gastric secretion induced with carbachol, histamine, and gastrin. Gut.

[3] Hutson WR, Roehrkasse RL, Wald A (1989). Influence of gender and menopause on gastric emptying and motility. Gastroenterology.

[4] Tuo B, Wen G, Wei J, Liu X, Wang X, Zhang Y, Wu H, Dong X, Chow JY, Vallon V, Dong H (2011). Estrogen regulation of duodenal bicarbonate secretion and sex-specific protection of human duodenum. Gastroenterology.

[5] Nie X, Xie R, Tuo B (2018). Effects of estrogen on the gastrointestinal tract. Dig Dis Sci.

[6] Zárate S, Stevnsner, T, Gredilla R. Role of estrogen and other sex hormones in brain aging. Neuroprotection and DNA repair. Front Aging Neurosci. 2017, 22;9:430. 10.3389/fnagi.2017.00430 PMC5743731 29311911

[7] https://doi.org/10.3389/fnagi.2017.00430

[8] Palomba S, Di Cello A, Riccio E, Manguso F, La Sala GB. Ovarian function and gastrointestinal motor activity. Minerva Endocrinol. 2011,36(4):295–310. https://www.minervamedica.it/en/journals/minerva-endocrinologica/article.php?cod=R07Y2011N04A0295 22322653

[9] De Villiers TJ, Pines A, Panay N, Gambacciani M, Archer DF, Baber RJ, Davis SR, Gompel AA, Henderson VW, Langer R, Lobo RA, Plu-Bureau G, Sturdee DW. International Menopause Society. Updated 2013 International Menopause Society recommendations on menopausal hormone therapy and preventive strategies for midlife health. Climacteric. 2013,16(3):316–37. https://doi.org/10.3109/13697137.2013.795683 10.3109/13697137.2013.795683 23672656

[10] Gursoy AY, Kiseli M, Caglar GS (2015). Melatonin in aging women. Climacteric.

[11] Walecka-Kapica E, Chojnacki J, Stępień A, Wachowska-Kelly P, Klupińska G, Chojnacki, C. Melatonin and female hormone secretion in postmenopausal overweight women. Int J Mol Sci. 2015,5;16(1):1030–1042. https://doi.org/10.3390/ijms16011030 10.3390/ijms16011030 PMC4307288 25569084

[12] Kato K, Murai I, Asai S, Takahashi Y, Matsuno Y, Komuro S, Kurosaka H, Iwasaki A, Ishikawa K, Arakawa Y. Central nervous system action of melatonin on gastric acid and pepsin secretion in pylorus-ligated rats. Neuroreport. 1998, 1;9(17):3989–3992. 10.1097/00001756-199812010-00040 9875741

[13] Sjöblom M, Flemström G (2003). Melatonin in the duodenal lumen is a potent stimulant of mucosal bicarbonate secretion. J Pineal Res.

[14] Reiter R.J, Tan DX, Rosales-Corral S, Galano A, Zhou X.J, Xu B. Mitochondria: central organelles for Melatonin's antioxidant and anti-aging actions. Molecules. 2018,24;23(2). pii: E509. https://doi.org/10.3390/molecules23020509 10.3390/molecules23020509 PMC6017324 29495303

[15] Klupińska G, Popławski T, Smigielski J, Błasiak J, Chojnacki J. The effect of melatonin on oxidative DNA damage in gastric mucosa cells of patients with functional dyspepsia. Pol Merkur Lekarski 2009,26(155):366–69. 19606675

[16] Pozo MJ, Gomez-Pinilla PJ, Camello-Almaraz C, Martin-Cano FE, Pascua P, Rol A, Acuña-Castroviejo D, Camello PJ (2010). Melatonin, a potential therapeutic agent for smooth muscle-related pathological conditions and aging. Curr Med Chem.

[17] Andersen LP, Gögenur I, Fenger AQ, Petersen MC, Rosenberg J, Werner MU (2015). Analgesic and antihyperalgesic effects of melatonin in a human inflammatory pain model: a randomized, double-blind, placebo-controlled, three-arm crossover study. Pain.

[18] Chojnacki C, Poplawski T, Klupinska G, Blasiak J, Chojnacki J, Reiter RJ. Secretion of melatonin and 6-sulfatoxymelatonin urinary excretion in functional dyspepsia. World J Gastroenterol. 2011,7;17(21):2646–51. https://doi.org/10.3748/wjg.v17.i21.2646 10.3748/wjg.v17.i21.2646 PMC3110928 21677834

[19] Chojnacki C, Popławski T, Blasiak J, Chojnacki J, Reiter RJ, Klupinska G (2013). Expression of melatonin synthesizing enzymes in Helicobacter pylori infected gastric mucosa. Biomed Res Int.

[20] Chojnacki C, Poplawski T, Blasiak J, Chojnacki J, Klupinska G. Does melatonin homeostasis play a role in continuous epigastric pain syndrome? Int J Mol Sci 2013,14;14(6):12550–12562. https://doi.org/10.3390/ijms140612550 10.3390/ijms140612550 PMC3709799 23771022

[21] Klupińska G, Stec-Michalska K, Chojnacki C, Wiśniewska-Jarosińska M, Walecka-Kapica E. Melatonin concentration in gastric juice and level of malondialdehyde in gastric mucosa of *Helicobacter pylori*—positive patients. Med Sci Tech 2009, 50(3): RA155–158. https://www.medscitechnol.com/abstract/index/idArt/881682

[22] Sugano K, Tack J, Kuipers EJ, Graham DY, El-Omar EM, Miura S, Hurama K, Asaka M, Uemura N, Malferthainer P. Kyoto global consensus report on *Helicobacter pylori* gastritis. Gut 2015; 64(9): 1353–1367. https://gut.bmj.com/content/64/9/1353. 10.1136/gutjnl-2015-309252 PMC4552923 26187502

[23] Bubenik GA, Konturek SJ. Melatonin and aging: prospects for human treatment. J Physiol Pharmacol. 2011, 62(1):13–9. https://www.jpp.krakow.pl/journal/archive/02_11/pdf/13_02_11_article.pdf 21451205

[24] Rohr UD, Herold J (2002). Melatonin deficiencies in women. Maturitas.

[25] Toffol E, Kalleinen N, Haukka J, Vakkuri O, Partonen T, Polo-Kantola P (2014). Melatonin in perimenopausal and postmenopausal women: associations with mood, sleep, climacteric symptoms, and quality of life. Menopause.

[26] Vakkuri O, Kivelä A, Leppäluoto J, Valtonen M, Kauppila A (1996). Decrease in melatonin precedes follicle-stimulating hormone increase during perimenopause. Eur J Endocrinol.

[27] Saha DR, Datta S, Chattopadhyay S, Patra R, De R, Rajendran K, Chowdhury A, Ramamurthy T, Mukhopadhyay AK (2009). Indistinguishable cellular changes in gastric mucosa between *Helicobacter pylori* infected asymptomatic tribal and duodenal ulcer patients. World J Gastroenterol.

[28] Klupińska G, Chojnacki C, Knopik-Dabrowicz A, Wojtuń S, Stec-Michalska K. Estimation of gastric mucosa morphological changes in subjects with asymptomatic Helicobacter pylori infection and family history of gastric cancer. Pol. Merkur. Lekarski 2004, 17 Suppl 1, 142–144. https://www.ncbi.nlm.nih.gov/pubmed/15603374 15603374

[29] Arabski M, Klupinska G, Chojnacki J, Kazmierczak P, Wisniewska-Jarosinska M, Drzewoski J, Blasiak J (2005). DNA damage and repair in Helicobacter pylori-infected gastric mucosa cells. Mutat Res.

[30] Ford AC, Forman D, Hunt R H, Yuan Y, Moayyedi P. Helicobacter pylori eradication therapy to prevent gastric cancer in healthy asymptomatic infected individuals: systematic review and meta-analysis of randomised controlled trials. BMJ 2014, 348 (may20 1), g3174–g3174. https://dx.doi.org/10.1136/bmj.g3174 10.1136/bmj.g3174 PMC4027797 24846275

[31] Werbach MR. Melatonin for the treatment of gastroesophageal reflux disease. Altern. Ther. Health Med. 14 (4), 54–58. https://www.ncbi.nlm.nih.gov/pubmed/18616070 18616070

[32] Klupińska G, Poplawski T, Drzewoski J, Harasiuk A, Reiter RJ, Blasiak J, Chojnacki J (2007). Therapeutic effect of melatonin in patients with functional dyspepsia. J Clin Gastroenterol.

[33] Celinski K, Konturek PC, Konturek S J, Slomka M, Cichoz-Lach H, Brzozowski T, Bielanski W. Effects of melatonin and tryptophan on healing of gastric and duodenal ulcers with Helicobacter pylori infection in humans. J. Physiol. Pharmacol. 2011, 62 (5), 521–6. https://www.jpp.krakow.pl/journal/archive/10_11/pdf/521_10_11_article.pdf 22204799

[34] Chojnacki C, Walecka-Kapica E, Lokieć K, Pawłowicz M,Winczyk K, Chojnacki J, Klupińska G. Influence of melatonin on symptoms of irritable bowel syndrome in postmenopausal women. Endokrynol. Pol. 2013, 64 (2), 114–20. https://journals.viamedica.pl/endokrynologia_polska/article/view/34290 23653274

[35] Siah KTH (2014). Melatonin for the treatment of irritable bowel syndrome. World J Gastroenterol.

[36] Jena G, Trivedi PP. A review of the use of melatonin in ulcerative colitis: experimental evidence and new approaches. Inflamm. Bowel Dis. 2014, *20* (3), 553–563. https://dx.doi.org/10.1097/01.MIB.0000436962.32164.6e 10.1097/01.MIB.0000436962.32164.6e 24247651

[37] Esteban-Zubero E, López-Pingarrón L, Alatorre-Jiménez MA,Ochoa-Moneo P, Buisac-Ramón C, Rivas-Jiménez M, Castán-Ruiz S, Antoñanzas-Lombarte Á, Tan D.-X, García J J. e. Melatonin’s role as a co-adjuvant treatment in colonic diseases: a review. Life Sci. 2017, 170, 72–81. https://dx.doi.org/10.1016/j.lfs.2016.11.031 10.1016/j.lfs.2016.11.031 27919824

[38] Harpsøe NG, Andersen LPH, Gögenur I, Rosenberg J (2015). Clinical pharmacokinetics of melatonin: a systematic review. Eur J Clin Pharmacol.

[39] Vural EMS, van Munster BC, de Rooij SE (2014). Optimal dosages for melatonin supplementation therapy in older adults: a systematic review of current literature. Drugs Aging.

[40] Bellipanni G, Marzo DI, F, Blasi F, Di Marzo A.  (2005). Effects of melatonin in perimenopausal and menopausal women: our personal experience. Ann N Y Acad Sci.

[41] Parandavar N, Abdali K, Keshtgar S, Emamghoreishi M, Amooee S. The Effect of Melatonin on Climacteric Symptoms in Menopausal Women; A Double-Blind, Randomized Controlled, Clinical Trial. Iran. J. Public Health 2014, 43 (10), 1405–16. https://www.researchgate.net/publication/278044343_The_Effect_of_Melatonin_on_Climacteric_Symptoms_in_Menopausal_Women_A_Double-Blind_Randomized_Controlled_Clinical_Trial PMC4441894 26060703

[42] Goya A, Terry PD, Superak HM, Nell-Dybdahl CL, Chowdhury R, Phillips LS, Kutner MH (2014). Melatonin supplementation to treat the metabolic syndrome: a randomized controlled trial. Diabetol Metab Syndr.

[43] Cardinali DP, Hardeland R (2017). Inflammaging, metabolic syndrome and melatonin: a call for treatment studies. Neuroendocrinology.

[44] Andersen LPH, Werner MU, Rosenkilde MM, Harpsøe NG, Fuglsang H, Rosenberg J, Gögenur I (2016). Pharmacokinetics of oral and intravenous melatonin in healthy volunteers. BMC Pharmacol Toxicol.

[45] Gooneratne NS, Edwards AYZ, Zhou C, Cuellar N, Grandner MA, Barrett JS (2012). Melatonin pharmacokinetics following two different oral surge-sustained release doses in older adults. J Pineal Res.

[46] Waldhauser F, Waldhauser M, Lieberman HR, Deng MH, Lynch HJ, Wurtman RJ (1984). Bioavailability of oral melatonin in humans. Neuroendocrinology.

